# Improving Equine Welfare through Human Habit Formation

**DOI:** 10.3390/ani11082156

**Published:** 2021-07-21

**Authors:** Jo White, Ruth Sims

**Affiliations:** 1 Human Behaviour Change for Animals CIC, Renhill, Mill Road, Barnham Broom, Norfolk NR9 4DE, UK; 2 Ruth Sims, School of Psychology, College of Health, Psychology, and Social Care, University of Derby, Derby, Derbyshire DE22 1GB, UK; r.sims3@derby.ac.uk

**Keywords:** equine welfare, equine behaviour, human–horse relationship, habit, human behaviour change

## Abstract

**Simple Summary:**

Human habitual behaviours are strong, long lasting and require less mental energy to perform than other behaviours, and these characteristics present an opportunity for developing pro-animal welfare habitual behaviours (PAWHBs) that benefit horses and donkeys. The routine nature of equine care lends itself to positive habit formation in people. This study addresses a gap in evidence and understanding of the impact of an intervention designed to develop PAWHBs in equine carers. People caring for horses and donkeys were asked to perform an enrichment behaviour with an equine in the form of scratching them in a consistent context linked to an existing routine behaviour. The findings indicated that linking new behaviours to existing routine behaviours can aid the development of pro-animal welfare habits. This also highlighted the role of the equine in triggering the new behaviour to be performed, the potential mutual benefit from engaging in the scratching behaviour for human and equine animal, and the positive emotions resulting from the action, which in turn promotes the repetition of behaviour. The findings mirror the theory of the habit loop: a cue to do a behaviour, the performance of the behaviour in response to the cue, and then the reward for doing the behaviour.

**Abstract:**

This paper explores the potential for interventions to develop pro-animal welfare habitual behaviours (PAWHBs) in people to improve the lives of animals. Human behavioural research indicates that opportunities exist to deliver lasting change through developing positive habitual behaviours. The routine nature of many equine care and management practices lends itself to habit formation and maintenance. This proof-of-concept paper aims to evaluate a theory-based intervention of developing and maintaining a PAWHB in people caring for equines. Qualitative research methods were used. A 30 day PAWHB intervention (PAWHBInt) of providing enrichment to an equine by scratching them in a consistent context linked to an existing routine behaviour was undertaken. Participants (n = 9) then engaged in semi-structured interviews that were analysed using thematic analysis, where the participants self-reported the outcomes they observed during the intervention. The study findings suggest that the PAWHBInt had a positive impact on human behaviour and habit formation. The research helps to address the dearth of evidence regarding the application of habit theory to equine welfare interventions and emphasised linking a desired new behaviour to an existing routine behaviour when developing PAWHBs. The research also highlights the role of mutual benefit for human and equine, and emotion in providing feedback and potential reward, supporting the link to the cue-routine-reward principle of habit theory.

## 1. Introduction

With the majority of the world’s horses, ponies, donkeys and their hybrids being domesticated animals utilised for work, sport, leisure and production, the human–equine relationship is central to equine welfare [1,2]. To promote and sustainably improve equine welfare, caregivers must ensure that they meet the welfare needs of the animals they are responsible for [3]. The human role and responsibility of caregiver in the human–equine relationship is increasingly recognised [1,2], with many countries around the world aiming to maintain and safeguard this by developing policies such as Duty of Care, Codes of Practice, guidelines linked to Animal Welfare legislation and frameworks such as One Welfare, which recognise the link between human and animal regarding wellbeing [4]. The conception of the Five Freedoms [5] or Five Needs (Animal Welfare Act, 2006) and, more recently, the development of the Five Domains [3] has provided frameworks to assess and measure welfare, while at the same time informing caregivers of the needs of the animals in their care [6]. The focus on ‘thrive not just survive’ [7] as a concept under the Five Domains illustrates the increasing attention being placed on the potential for a positive relationship and bond between human and equine. This emerging emphasis has created an opportunity for those working in equine welfare to utilise evidence-based approaches from human behavioural science to understand both the behaviour of the human and what positive behaviour change could improve equine wellbeing [8]. This last point has provided the focus for this proof-of-concept study, which aims to explore how to develop and maintain simple sustained pro-animal welfare behaviours through the formation of positive habits among equine caregivers.

Animal care requires the routine performance of behaviours by a person to maintain an animal’s physiological and psychological welfare [7,9,10]. This is very evident in the management of horses and donkeys, with reference to routine management prominent in publications, literature and training resources across the equestrian sector [11]. One of the root causes of animal suffering and neglect is behavioural action or inaction on the part of human animals [8]. For example, a behavioural action that results in a negative welfare outcome for the animal could be hitting or whipping them, and a behavioural inaction could be not providing fresh, clean water for the animal. Poor or compromised animal welfare is frequently connected to the incorrect execution of, or failure to perform, routine management behaviours [12,13]. Routine equine care presents an opportunity for professionals to deliver positive interventions that result in improved animal welfare, which may be achieved through the formation, maintenance and change of habitual behaviour in the human animal [14] around equine care routines.

Routine has been conceptualised as “repetitive patterns of interactions” [15] (p. 1) consisting of separate but interconnected habits. In the context of equine care, this might relate to routines around checking animals’ physical health or cleaning their environment (e.g., stable, field). Research into routine and habit formation is attracting growing interest, especially in terms of explorations of habit formations potential to deliver long-term behaviour change [14,16,17]. Studies have also indicated the importance of habits as part of the daily behaviours people perform: one indicates that daily behaviours are habitual in nature in 35% of cases and another in 43% [18]. However, currently, there is little research regarding the role of habit formation in routine equine care and welfare or its potential for developing sustained positive behaviours in people while addressing negative ones [19].

Both the study of habit (Aristotle 384–322 BC) and the earliest book of horsemanship referring to routine care (Xenophon circa 355 BC) date back to ancient Greece. Barandiaran and Di Paolo suggest that the classical authors interpreted ‘habit’ as developing from “repetition” or “custom” into something that could be called “second nature” [20] (p. 5). Translations of Xenophon make reference to actions indicative of routine and repetitive behaviours, including cleaning out litter every day and the horse and rider practising movements learnt during training [21]. Historically, psychologists such as Dewey (1922) and James (1890) have provided studies of habit and routine [15]. The *Oxford Dictionary of Psychology* defines habit as “A disposition to behave in a particular way, or an established practice or custom”, and, in the context of learning theory, as “a learned behavioural response associated with a particular situation, especially a response that has been subject to reinforcement or a conditioned response” [22] (p. 328). These definitions emphasise a link between habit formation and classical (Pavlovian) and operant (Skinnerian) conditioning, where new behaviours are developed through an association between stimuli or cue and response or action [23] (e.g., a yard manager trains a groom to pick a horse’s feet out on collecting them from the field, the cue is bringing the horse from the field, the response is cleaning the hooves). However, in contrast to conditioning, habit is slow to develop and change [24] (e.g., positive and proficient equine handling skills take a long time to become automatic in nature). While the focus on habit wavered for some years owing to the rise of theories such as behaviourism, focusing on conditioning rather than habit [20,25], more recently, there is growing interest within psychology [26]. There has been a recognition of the potential of habit’s properties in developing positive behaviours, as is apparent in the human health sector, and in the development of models such as the Theory of Interpersonal Behaviour [27]. This growing interest highlights that there is an opportunity to utilise habit to create sustainable improvements to equine welfare.

In this revival of interest, habit has been described as linked to learning, as a series of behaviours that becomes an automatic response to particular cues and has the role of achieving specific end goals [28] (e.g., a person moves from learning how to practically assess a horse for pain with the goal of safeguarding wellbeing, to the behaviour becoming automatic and cued when in the presence of a horse). Contrastingly, Neal et al. emphasise habit as being triggered by contextual cues, with goals being insignificant, suggesting that, while habit may not be receptive to altered motivation, a change in the contextual cue could be effective as an intervention for habit change [29] (e.g., the habit of feeding a horse may be related to the contextual cue of a certain time, place and/or procedure, with changes to one or more of these contextual cues eliciting a change in human behaviour). However, there is general agreement that habit may start by pursuing a goal using active conscious reflective thought (e.g., the goal of reducing the weight of a horse or donkey), before moving towards contextual cues that trigger an automatic response to perform the behaviour (e.g., the person is cued by the presence of a weighing scale in the feed room to weigh the feed they are providing). It has also been noted that a person is more likely to repeatedly perform behaviours that are rewarding, such as riding their horse out on a hack to enjoy the reward of spending time together in nature while exercising the horse to lose weight [26,30].

Duhigg, referring to the ‘habit loop’, suggests that habits work on a ‘cue–routine–reward’ basis, which, with repetition, becomes automatic [31]. Habit is described as the consistent repetition of a behavioural action in the company of established contextual cues that strengthen the behaviour [32]. Once the habit is in place, the presence of the relevant context should result in a reduction in the cognitive energy and input necessary to perform the behaviour, saving the brain resources that can be utilised in other ways [19,33]. These features of habits in general are also characteristic of routine behaviours related to equine husbandry that are consistently and repeatedly performed in the same situation with little conscious thought and requiring no active decision-making, such as the feeding of a horse in their field or stable as part of a morning routine. The potential benefits of being able to perform pro-equine welfare related behaviours automatically means they are more likely to be performed than those behaviours requiring reflective conscious thought and motivation.

Fogg discusses how forming many ‘tiny habits’, rather than focusing on major changes that require high levels of motivation and willpower, can help change and maintain behaviours [34]. He examines the value of linking the desired habit to a current routine (e.g., before or after making a cup of tea I will do [insert behaviour to be linked]) in increasing the likelihood of the behaviour being performed habitually (e.g., after bringing my horse in from the field I will check their body for injuries). Similarly, other research shows the need for an established, stable contextual cue for habit formation [17,30,32]; with a regular routine acting as the trigger for the habitual behaviour [34]. This is supported by research examining prospective memory, which demonstrates that activities connected to an established routine are easier to remember [35]. This approach could therefore be applicable in habit-formation or change interventions in equine welfare; owing to the regular, consistent nature of many animal husbandry routines, a variety of options are available with which to connect a new behaviour. For example, grooms or yard staff working with horses tend to perform the same care routine every day, which could present an opportunity to link a new pro-animal welfare habitual behaviour (PAWHB) to the existing routine.

Gardner suggests that, where habit and intention to perform behaviours come into competition, the behaviour performed tends to follow habit rather than intention [30]. Therefore, if PAWHBs can be successfully developed they could present strong and sustainable routes to improving the welfare of animals. For example, in handling horses and donkeys training interventions are frequently used to improve the way people perform actions, but sustainable long-term success will depend on whether the desired behaviours are established as habits. Persons who have already developed habitual behaviours that are contrary to welfare, such as hitting non-compliant horses or donkeys, are more likely to continue performing the established habit than to switch to a recently trained intentional behaviour unless the positive handling behaviours are repeated in a consistent context such that they become automatic and habitual. The existence of conflicting habits could explain why intention-focused behaviour change interventions are not always successful [19]. In addition, behaviour change that focuses on intention also relies on conscious mental energy, motivation and willpower, which strongly formed habits do not [19]. It is postulated that the formation of PAWHBs has the potential to sustainably improve equine welfare through the development of simple and accessible behaviour change interventions that can be utilised in the UK and overseas by professionals such as vets and NGO workers working to improve the health and wellbeing of equines. To date there is little research relating to the application of habit to behaviour change interventions in an animal-welfare setting, as enquiry has tended to focus on changing attitudes, beliefs and perceptions and exploring planned behaviour. It is the aim of this proof-of-concept study to begin the process of addressing this gap in knowledge through evaluating the following research question: can a behaviour change technique of implementing a pro-animal welfare habitual behaviour intervention (PAWHBInt) support the development or maintenance of a PAWHB?

It should be noted that the cessation of negative animal welfare habitual behaviour is outside the scope of this study, but presents possibilities for future research.

### 1.1. The Study 

This paper covers the qualitative analysis component of a mixed-methods study to examine the application of a habit intervention covering human–equine interactions linked to animal welfare. It is intended that this will provide a useful starting point for future research, increased understanding and practical application. The research was designed to be a mixed-methods study to enable an understanding of a complex issue, but it was not possible to recruit a naive population of participants and therefore the quantitative data were used only to select participants for the qualitative inquiry. The target group of equine carers was selected for this study as an accessible population demonstrating many routine behaviours appropriate to this study. The behaviour to be performed must meet the following criteria: it must be positive, simple, easy to perform and easy to fit into an existing routine [34]. The selected behaviour was to routinely scratch an equine, which mimics equine mutual grooming and could enhance the human–animal social bond [36]. Its advantages for equine welfare are that it has the potential to provide social stimulation and enrichment for the animal, as social isolation has been identified as a welfare concern [37]. It presents a straightforward behaviour that can be linked to a consistent context, routine and reward. Scratching an equine is posited to be viewed by participants as a positive behaviour; it is hypothesised that it may act as an emotional reward for the human, who perceives they are doing something positive for the horse or donkey. It is also potentially positive for the equine, who benefits from the interaction [2]. Once preliminary research is undertaken to understand the application of habit theory in an equine welfare context, more challenging habitual behaviours could be examined in relation to habit formation, maintenance and change.

### 1.2. The Aim of the Study

The aim of the study was to evaluate a behaviour change technique using a PAWHBInt to develop and maintain the performance of a PAWHB of scratching an equine once a day for a minimum of three to five minutes in a consistent context linked to an existing routine behaviour. The intervention designed to encourage the performance of the described behaviour used evidence-based theories and models of behaviour change and habit formation, including a 30 day intervention period, training, an action plan and a self-monitoring diary. A study by Lally et al. modelling habit formation in everyday life, found that it took between 18 and 254 days for the connection between repetition and habit strength to become automatic for participants; the average was 66 days [32]. To reflect these findings and comply with the study time constraints, the PAWHBInt was performed every day over a 30 day period, which is also supported by anecdotal evidence of interventions or ‘challenges’ using a 30 day timeframe [38]. Basic training materials were provided (see Appendix A Part A) to ensure that participants understood how to undertake the intervention, how to scratch the equine and what the possible benefits of the behaviour were. A lack of knowledge impacts on a person’s capability to perform a behaviour [39] and has been cited as one of the main perceived causes of poor equine welfare in Great Britain [37]. The materials included ethical considerations to protect the equines involved and avoid any negative effects. Each participant was provided with a simple action plan to complete (see Appendix A Part B) with the aims of aiding implementation intentions [17,40] to perform the scratching activity, ensuring consistency, assisting in initially establishing the behaviour and linking the new behaviour with an existing routine [17,34]. The plan to link the behaviour to an existing routine was intended to reinforce the association between the cue and the action with each repetition in order to increase the automaticity of the behaviour [40]. Participants identified within their daily routine a contextual cue to which they linked the scratching behaviour (e.g., after picking hooves out). The plan included a reminder (e.g., a visual cue at the stable/field) to provide an additional cue and aid to memory; this is important in developing new habits, particularly at first, but its impact is said to weaken with time [17]. The participants were required to complete a daily self-monitoring diary (SMD) (see Appendix A Part B) for the 30 days of the PAWHBInt. This covered whether they had performed the scratching behaviour or not and allowed for any comments, observations or reflections. Its purpose was to motivate, positively reinforce and reward the performance of the behaviour through the participants’ reflection on the outcomes (e.g., the animals’ reactions to the interaction). Additionally, it helped to ensure that the behaviour was performed consistently in relation to the context [19]. Analysis of physical activity and eating behaviour research have indicated that interventions are significantly more effective when incorporating self-monitoring [41].

## 2. Materials and Methods

As stated above, a mixed-methods design was used. The quantitative data captured through surveys (n = 48) (Appendix A Part C) informed the recruitment and selection of nine participants for qualitative analysis, only the qualitative data will be discussed in this paper. All the participants completed the PAWHBInt prior to undertaking the semi-structured interviews. A thematic analysis [42] was undertaken to provide insight into and understanding of the impact of the PAWHBInt on the participants and the study research question. Qualitative inquiry was selected as it is a useful way of exploring an issue where information is lacking, as in the case of habit formation to improve equine welfare [43,44]. The study was ethically approved and met Derby University’s: Good Scientific Practice Policy and ethical review procedure.

Forty-eight participants were recruited via opportunistic sampling, meaning they were readily available and accessible for the study; posters on equine social and online media, together with emails sent to equestrian sector contacts were used (Appendix A Part D). To avoid demand characteristics and bias, the participants were not informed that the research was a study of human habit formation, and no incentives were used for participation. The participants were sampled to represent a population of primary carers of equine animals. The inclusion criteria consisted of being a UK-based adult (aged >18 years) who cares for an equine animal and has permission to undertake the PAWHBInt with the animal. Participants were excluded if they or their animal were suffering an injury or illness that would place them at increased risk. To provide an adequate sample size, all participants who met the inclusion criteria were included. The sample (n = 48) consisted of 2 males and 46 females, whose ages ranged from 22 to 71 (mean age of: 49 years, SD = 12.065); 90% (43) owned and cared for the equine animal, while the remainder were involved in caring for the animal but did not own them. The participants were given a briefing outlining the study approach and a consent form (Appendix A Part E) stating that their data would be protected and kept confidential. They were asked to create a unique participant reference code to maintain their anonymity and were provided with information on how to withdraw should they no longer wish to participate. The participants’ information was kept in a locked office, data were protected by a password and participants were sent a web-link to the first survey (before the PAWHBInt) (Appendix A Part C). The participants were given the intervention materials, including a standalone PowerPoint slideshow for training purposes (Appendix A Part A), an action plan for completion prior to starting the scratching activity (Appendix A Part B) and an SMD form for daily completion during the PAWHBInt (Appendix A Part A & B). Online surveys were completed before, immediately after and 30 days after the PAWHBInt and were used to select participants for interview. The surveys were part of the quantitative study and will not be discussed in this paper. Nine (n = 9) participants undertook the audio-recorded individual semi-structured telephone interviews (secure line) conducted by the Primary Investigator (PI), which utilised an interview schedule of questions (Appendix A Part G). Semi-structured interviews were used to explore the issues experienced by participants. Semi-structured interviews give the participant the chance to discuss their experiences in detail through the use of open questions [45]. The sample size met requirements of achieving saturation through the recurrence of themes, which enabled the research question to be answered [46]. To identify and select suitable participants who represented different experiences of the intervention, purposive sampling was employed using the quantitative data set, which covered:

1. Participants who developed the PAWHB and self-reported as developing a strong habit when surveyed immediately after the intervention and again in the follow-up survey 30 days later;
2. Participants who developed the PAWHB and self-reported as developing a strong habit immediately after the PAWHBInt, but had not sustained this when surveyed again in the follow-up;
3. Participants who self-reported as not developing the PAWHB following the PAWHBInt;
4. Participants who self-reported as already performing the PAWHB.

Participants identified as suitable for interview were approached by email and given the option of declining. The participants were numbered P1 to 9, and were subsequently referred to by their numbers (e.g., P1 stated: “…”). All information that could identify the individual was removed. The recorded interviews took between 30 and 60 min to complete, and followed the interview schedule of questions (see Appendix A Part G): the participants were asked about undertaking the PAWHBInt, about any barriers to the activity, and for positive and negatives comments; how they felt regarding the activity and what impact it had on them; and what if any reflections they had on habit. On completion of the data collection, each participant received a written debrief email explaining the objectives, concluding the data collection phase of the study, and inviting comments (see Appendix A Part F). The interviews were then transcribed verbatim for analysis.

A thematic analysis [42], including the theory-led six-phase process for analysing the participants words and language, was used in the interviews to provide insight into the research question. This type of analysis seeks to identify patterns of meaning in the data. In the familiarisation phase (phase one) the interviews were read and notes made. Initial coding (phase two) was then carried out on two interviews to aid the generation of key themes by examining the meaning and repetition of phrases, concepts or patterns [47], and utilising an inductive approach. The units of analysis were phrases, sentences and paragraphs that related to the identified codes. An inductive and deductive analysis was then applied to all the remaining interviews by examining the identified codes and marking up the themes and language that interviewees used that had resonance to habit formation theory. This was to assist in identifying the main themes (phase three). A semantic approach was used to classify and review themes according to the explicit, overt meaning of the data, with a latent approach then applied in relation to habit formation theory and the links between themes (phase four). These approaches were important to enable the cross-referencing of the themes generated with evidence-based human behaviour theory. The main themes were then defined and named (phase five), in preparation for being reported (phase six).

## 3. Results

Data from nine semi-structured interviews with experimental group participants were evaluated using a thematic analysis in order to assess the impact of the PAWHBInt in forming and sustaining a PAWHB in a consistent context linked to an existing routine behaviour. The data generated five themes that were considered pertinent to the research question (refer p. 8): ‘habit or conscious thought’, ‘role of routine’, ‘animal feedback’, ‘change and intention’ and ‘research insights’; in addition, a number of sub-themes and links between themes were elucidated. The themes provided insight into the PAWHBInt, the participants’ experiences, the PAWHB, and the study research question. The study included individuals who already undertook the scratching behaviour and those who did not, providing greater insight into existing habit and the formation of new habit through the analysis. Table 1 explains how the themes related to the research question.

### 3.1. Theme 1: ‘Habit or Conscious Thought’ (Including Sub-Themes: Developed and Learnt; Context)

The theme was generated from participants’ responses involving language reflecting the characteristics of habitual behaviour (e.g., second nature, automatic) and references that participants made to developing and performing the PAWHB automatically, either as an existing behaviour or as one that occurred during the intervention, subsequently, or not at all. It examines the meaning of participants’ comments regarding the performance of behaviour habitually or with conscious thought, together with changes they made or intention shown to adapt their behaviour as a result of the PAWHBInt.

Some participants’ use of figurative speech and similes suggested an action that was routine, quotidian, unconscious or instinctive. It included references that are widely accepted in British society as relating to habit (e.g., making a cup of tea). This was interpreted by the Primary Investigator (PI) as meaning that the PAWHB was already an established habitual behaviour among these participants.

P3

… it comes as naturally as making a cup of tea in the morning.

P8

I have always had a habit of scratching her—that is how I interact with her. I hadn’t thought of it as a conscious thing, it is more second nature.

When asked how and why they developed and learnt the behaviour or other behaviours, three participants used language that indicated it was part of their routine from childhood, something they had learnt from their parents and had always done. The words used suggested that the behaviour may be a conditioned response and habitual in nature (e.g., “ingrained”). There were repeated references to routine, in terms of both past and present, with a sense of repetition over time indicative of a long-term habit.

P1

… maybe it was ingrained in me as a little girl but it’s like that, something that I have always done as part of my routine and so it has become part of his routine.

Participants who developed the PAWHB during the study used explicit language indicating a change from a conscious performance of the behaviour to an automatic one. A number used reflective language (“I think”) when describing how the behaviour had changed during the study time frame.

P6

When I visit the donkey now I suddenly realise I am scratching him in the same spot that we established over the 30 days. It has moved from being a conscious thing to an unconscious one that means I don’t realise I’m doing it.

Others appeared to move back and forth between conscious and automatic states, depending on other factors or contexts that appeared to result in them being more mindful of performing the behaviour, such as time constraints or attempts to take a more consistent, systematic approach. This suggested that, when there were additional factors to consider, conscious mental energy was required to think about the activity.

P4

I was conscious I was doing it systematically. I do tend to do quite a lot of scratching all over their body anyway and I do that without realising but because I was consciously trying to do this in the same way every day I was conscious of what I was doing … I think it was a mix of automatic and intentional behaviour.

Two participants made reference to carrying out scratching behaviour prior to the study, but in a different context and place on the equine animal from those they undertook for the study. The words used suggested that the existing scratching behaviour was habitual, whereas the new behaviour was not, and mental energy was involved in thinking about performing it.

P5

No, it definitely wasn’t automatic because I had to think about doing it …It would be automatic if he just reversed into me and I scratch round his tail … that would be automatic, but this wasn’t at any point.

### 3.2. Theme 2: ‘Role of Routine’ (Including Sub-Themes: Action Plan, Memory, Consistent Context, Cue, Reminder, Repetition, Mutual Benefit, Emotion)

Routine can be considered to be a core component of habitual behaviour and its formation [14]. This theme was generated from the pattern and frequency of comments made by participants about routine generally as well as in relation to the PAWHBInt action plan made to link the behaviour to an existing routine. It covers the participants’ indications of the value of routine in aiding memory and providing a consistent context and cue for the regular enactment of the behaviour, allowing the participant to move from conscious thought to automatic behaviour.

Participants used words that indicated a link between the scratching behaviour and an existing routine in aiding memory. This was interpreted to mean that the routine acted as a cue and reminder to perform the behaviour through the connection to an activity performed systematically and consistently, suggesting another factor in establishing habitual behaviour: repetition. Participants reflected on how using an existing routine in this way made it easier to perform the scratching behaviour, a number crediting this with ensuring that they did not forget to perform the behaviour and with successfully embedding it into the existing routine. The language used indicated that the behaviour became automatic, suggesting a change towards establishing the PAWHB.

P2 (routine chosen—grooming)

… I felt if I did it at that time there was no chance of me forgetting. There was once or twice when I was about to put all his grooming stuff away and I thought I haven’t done it. Whereas I think if I hadn’t included it with a regular activity I might have forgotten.

P4

I had to make it a systematic approach and it just became that I incorporated into daily routine after a few days.

Participants who had made reference in their interview to the behaviour being automatic also talked about the scratching behaviour being part of their routine. They provided details of the routine itself and how scratching was incorporated within this, thus illustrating the link between habit and routine:

P1

It is something I do routinely with him, you know you go in and give him a bit of a scratch—He has a routine he knows I go in and do his bed and then it’s grooming time and scratch time.

P3

Pretty much, my routine would be, we have seven of them, go in, pat him, put head collar on, scratch him pick his feet out and turn him out.

There was also reference to the value or mutual benefit of having a routine, for both the person and the equine. Some participants reflected on the emotional impact of the routine, which made them feel calmer.

P6

I quite like routine; it was nice to establish a routine and that made me feel calmer in myself that there was a pattern of behaviour being established. It was part of establishing a better routine of working with the donkeys.

### 3.3. Theme 3: ‘Animal Feedback’ (Including Sub-Themes: Cue, Mutual Benefit, Reward, Motivation, Emotion, Bonding, Context, Repetition)

The theme of ‘animal feedback’ was generated by the participants’ references to the equine animal’s responses to the scratching behaviour and the impact of these on establishing and maintaining the PAWHB, or in some cases not establishing it. Some of the participants indicated that the feedback they received may have resulted in the equine effectively acting as an additional cue in triggering the scratching behaviour. Participants also made reference to mutual benefits for both the equine and human, which in habit theory suggests links to a reward; additionally, these can aid motivation to perform the behaviour while it is new.

Participants made reference to the equines’ behaviour and to their own interpretation of this behaviour as feedback relating to their performance of the scratching behaviour (e.g., whether they were scratching the animal in the preferred place or not). They also discussed their perceptions of what the animal wanted in relation to the interaction. The PI inferred from the language used by the participants (e.g., “they all demand it”) that this feedback or communication by the equine might act as an additional cue.

P1

Yes, even as far as I get the grumpy face and he will move and inch himself around until I get the right bit. He’s incredibly interactive.

P7

The trigger now is that they all demand it. You really have to stop poo picking, have a little scratch. So I don’t even have to initiate it now, it’s just whoever I am next to wants a cuddle, wants a scratch.

The perceived animal feedback appeared to link to the participants’ reflections on how they felt emotionally and the importance they placed on bonding with the animal. Positive language was used to describe both how they felt (e.g., “I loved”; “good fun”; “enjoyed”; “happy”) and how they perceived the animal’s feedback, with some participants using words suggesting that they were interpreting the animal’s thoughts from their behaviour. There were indications that these perceptions may have been acting as a mental and physical reward for the participants. The words used suggested that the response evoked by touching the animal was interpreted as representing the animal’s enjoyment of the person’s actions, resulting in the positive emotions reported by the participants, which encouraged them to repeat the behaviour.

P1

It is quite emotional because the bond is obviously there and the fact that he is talking to me letting me know how he wants it and also letting me know when I got it wrong, it is like a full on conversation in the sense that you’ve got that kind of bond with an animal.

P2

I loved doing it because I like being with him, touching him, grooming him and some days his reaction was more marked than others and when you could see that he obviously had an itchy place and his lips were twitching and it was quite good fun to see it. You could almost read their thoughts—ooh this is lovely keep scratching.

In contrast, two participants (P5 and P9) commented on the lack of feedback from the equine animals. They referred to negative feelings and emotions, using language suggesting a monotonous and effortful task (e.g., “a little bit mundane”; “a bit of a chore”; “‘gotta stick with it, gotta do it!’”) and signifying that the participants relied on willpower and motivation, rather than the reward of bonding with the equine animal, to continue the behaviour.

P5

Emotionally because I wasn’t getting anything back from him I didn’t feel any bonding going on between us, it almost felt like I was annoying him at some points.

P9

She made it very plain that scratching is something she and the other horse do and she and I interact differently.

… it was just we got to about day 5 or 6 and I realised it wasn’t going to be her favourite thing but having started we carried on.

The sub-theme mutual benefit was generated based on the participants’ words that linked animal feedback and emotion to a perceived mutual enjoyment of the scratching activity by human and equine, highlighting the importance of two-way benefits. Additionally, some of the participants used the scratching behaviour in other contexts to calm and provide reassurance to the animal during stressful experiences (e.g., first show, dental treatment), which benefited the participant directly.

P4

… important to do something that you think is going to be of benefit and it’s good if it benefits the owner as well because it’s got to be a two way thing.

P6

I really enjoyed it and so did the donkey—the donkey approached at the time that I routinely did the scratching…anything that he enjoys I enjoy.

### 3.4. Theme 4: Change and Intention (Including Sub-Themes: Cue, Context, Emotion, Mutual Benefit)

This theme was generated from the participants’ responses indicating support for the PAWHBInt resulting in the maintenance or development of a PAWHB. In most cases, this concerned the scratching behaviour only. However, in a few cases, the participant reflected on the equine feedback and what they perceived the animal enjoyed, and planned to undertake a future behaviour centred around that.

The participants who already undertook the PAWHB activity used language indicating that they assumed they would continue to do the behaviour long term, partly as a result of the animal’s feedback and cueing to continue and partly as “something I do as second nature” (P1).

P1

I don’t think he’d let me stop it. The way that he is he knows he has me wrapped round his little finger, the way the scratching happens.

P3

… scratching until I die.

While some of those who developed the behaviour during the study used words suggesting intention to continue performing it and using elements of the intervention (e.g., P4: “I think I will continue as I think it has benefit”), others said that they were not performing the behaviour as frequently, but mentioned doing it automatically when in the same context as used in the study (e.g., scratching the animal at the same time, place, part of their body; linked to the existing routine). In addition, participants talked about learning more from the animal’s behaviour about their individual preferences regarding being scratched in different places on their body, which they were now enacting.

P6

Yes, since the end of the 30 days I have done it less regularly but I am still routinely doing it, and less consciously but particularly with the donkey I worked with, when he first comes to the fence I spontaneously reach for the back of the ear which was the spot we established. So I think it has become an unconscious thing now.

The participants who had a negative experience also communicated that they had reflected as a result of the study and intended to either do the scratching behaviour or increase their “quality time” with the equine animal.

P5

Daily quality time for the two of us … it did possibly make me think more about a pleasurable sort of contact for him.

Others appeared to consider that the benefits of performing the behaviour provided an incentive to continue doing it (e.g., mutual grooming for an animal kept on their own; the ease and value of routine; spending quality time with the animal). A few participants referred to intending to carry out, or actually carrying out, the PAWHB with other equine animals and made links to the communication of positive emotions.

P8

… I have also started doing it with my other mare and it hadn’t even occurred to me that I have started doing it with her. She’s retired and it’s my way of saying hey I don’t ride you anymore but I still love you.

### 3.5. Theme 5: Research Insights (Including Sub-Themes: Human-Animal Interaction (HAI), Plan, Reminder, Training Material, Cue, Reward, SMD, Emotion, Motivation, Memory)

This theme was generated from insights into participants’ reflections, including those relating to the PAWHBInt and the study design, together with an understanding of the participants’ beliefs in relation to HAI and their possible biases relating to animal welfare.

The participants’ language indicated that, overall, the action plan was positively received, being easy to carry out, and was regarded as a successful element of the intervention. The plan included the option for participants to use a reminder for the scratching activity; for those that used the reminder, reference was made to it working initially and then no longer being needed.

P6

Yes I felt it was successful. A plan was important.

P7

I think it was successful for me and [for the equine]. And it was certainly easy.

P6

… I think because it was the first thing on my list I just did it and after a few days the reminder in the kitchen was less impactful.

It has been highlighted that participants P5 and P9 indicated that they did not scratch the equine animal where the animal preferred it, as stated in the training material (i.e., “If you have not scratched your horse, pony or donkey before you need to find their ‘sweet-spot’—the place where they enjoy being scratched the most”—see Appendix A Part A), but had used the training material example instead. This approach may have impacted on the feedback from the equine animals, which in turn influenced the participants’ cue, reward and motivation to do the behaviour.

P5

I stuck with the shoulder/base of the neck area, just to see whether he would—he stood there, sometimes he was a bit fidgety but you could see that he wasn’t. I was expecting more from him to be honest because he is such an itchy scratchy kind of guy.

P9

I don’t wither scratch, I scratch round her face. I think because it’s not what other horses do to her she therefore thinks it’s appropriate.

The language used by the majority of the participants suggested that they found the self-monitoring diary (SMD) easy to complete; indeed, some found it useful and enjoyable, which aided in reflecting on the activity. Others indicated that it was not useful, some because the response they received from the equine animal was the same as it always was, others because they found commenting on their own reactions challenging. A few made reference to it being something else they had to remember.

P6

Helpful for recording what was being done and the donkeys’ reactions. Slightly less helpful in recording my own reactions. I naturally record how I had perceived the donkey had felt that day and I would suddenly think I needed to record how I had felt.

P7

I found it really helpful. I worried that I was maybe giving too much information … but actually in the end it was like I was just doing it for me and I have kept my copy because I found it interesting the things I was saying and it was making me think. I thought it was really fantastic, I really enjoyed it.

P4

I suppose to some extent trying to remember to fill in the diary was an added job but it didn’t take that long so it was fine.

Five of the participants expressed a strong interest in the benefits of positive human–animal interaction (HAI). This sub-theme should be considered in the context of the impact this attitude may have on the study, depending on whether these participants are typical of the equine carer population or are skewed towards pro-animal welfare behaviours because of their interest in HAI. Participants referred to a long-term belief in HAI and considered that anyone connected with animals should have an appreciation of it; in reflecting on the study, they reiterated their beliefs in it.

P3

… I have always believed in HAI if you haven’t got that there’s no point in having animals.

P6

… I think it was a confirmation of how I feel—I’m convinced HAI is very important and participation in this has confirmed that.

## 4. Discussion

The impact of habit has been recognised in other areas, such as health, fitness and information technology [24], with the direct extent of its influence said to be equivalent to “cognitive, motivational and affective factors” [48] (p. 384). As such, it presents an opportunity to develop interventions that deliver improvements and address some of the key issues currently facing equine welfare, while at the same time enhancing human–equine interactions and relationships. This study aimed to evaluate whether a behaviour change technique of implementing a theory-based PAWHBInt was able to impact upon developing or maintaining a PAWHB. Evidence for pre-existing behaviours of this type was apparent in the participants, alongside the development of, change to and intention to perform a new PAWHB. The value of linking the new, desired behaviour to an existing routine was explored and observed to have strength in an equine-care setting.

### 4.1. Key Findings

The study provided evidence supporting the presence of PAWHBs in the participants. The interviews provided rich qualitative data and generated themes that support habit theory, particularly in relation to the role and workings of the ‘habit loop’ (cue–routine–reward) [31] in an applied practical setting. They also suggested that the PAWHBInt had an impact on the participants and their behaviour, with indications of positive change.

### 4.2. Implications for Findings

The study findings relating to pre-existing PAWHBs, together with the indications that behavioural change, or intention to change, was evident in relation to the intervention, supporting the need for further work examining the potential use of habit formation and change in equine welfare.

As outlined, the study data indicated that the majority of participants perceived that they were performing the scratching behaviour automatically every day; therefore, it was already habitual (theme ‘HorCT’). This is an important finding, as it provides evidence of the presence of routine habitual behaviour in an animal-care setting, which is the starting point for further work in this area. However, to understand this further and to examine the impact on the PAWHBInt findings, it is worth considering the language used during the interviews surrounding HAI to determine whether the participants were predisposed towards pro-animal welfare behaviour.

Of the participants who reported that they were not performing the PAWHB automatically before the study, a few stated that they remained conscious of their actions when doing the behaviour depending on the context (e.g., the availability of time), with some moving between conscious thought and unconscious–automatic action. This was also observed by Fleig et al., who found that some participants reported the activity becoming automatic and mentioned “increased awareness and attention” [49] (p. 122). This identifies an area for further inquiry regarding movement between conscious and unconscious behaviour in a way that elicits reflection and consideration of an existing habit, particularly in terms of whether that could be utilised to prompt change to improve equine welfare.

For those for whom the PAWHB became automatic during the research, the study design, involving repetition, is thought to be a contributing factor. In addition, support for the use of an action plan to help participants perform the behaviour in a consistent context linked to an existing routine [30,32,34,50] was provided by the qualitative analysis (‘RofR’), with participants reporting that the activity became easier owing to the link with the routine. This agrees with previous studies that indicate that, through repetition and consistent contextual cues, a desired behaviour can become automatic, requiring less cognitive energy [24,40,50]. It is posited that the repetitive nature of many equine-care activities presents an opportunity for interventions utilising repetition linked to existing routines to establish a PAWHB. In addition, using less cognitive energy may be advantageous if it aids long-term performance of tasks and enables the use of conscious thought in other ways that benefit the equine.

As the new behaviour of scratching the equine animal became embedded in the participants’ routines over the 30 days it became easier to remember, as indicated by the sub-theme ‘memory’, which is endorsed by studies examining prospective memory [35]. Previous research into health [49] found that some cues aided memory in terms of performing a required activity and that an action plan incorporating habit formation using activity-based or existing routines as cues may help in reducing demands on memory, which was a finding of this study. There is again, an opportunity to utilise the routine nature of animal care to link new desired behaviours with existing routines.

The theme ‘animal feedback’ is particularly noteworthy. This was generated from participants’ comments that suggested that the equine was acting as an additional cue to perform the behaviour; the participants indicated that the animal appeared to communicate their enjoyment or otherwise of the scratching interaction. This may indicate that the PAWHB had become a two-way action between the human and the equine, with each acting as a contextual cue for the other [51].

Feedback from the equine might also have acted as an emotional reward for doing the behaviour and as a motivator during the intention stage of habit formation. This links to the sub-theme of mutual benefit, where participants and the equines both benefited from the interaction either directly (e.g., a closer bond) or indirectly (e.g., reassurance for the animal during dental treatment); in turn, this acted as an incentive to continue doing the habitual behaviour, as cited in the ‘habit loop’ [31]. The interviewees who indicated that the behaviour was not automatic referred to limited or absent feedback from the equine animal and a lack of connection or bond. It is postulated that this may have resulted in a lack of motivation to do the behaviour and a lack of reward for performing it, which effectively breaks the feedback chain in the habit loop of cue–routine–reward. This is important to consider in the context of an animal welfare intervention, as without a relevant motivator to aid in consciously moving the behaviour from intention to action, and a suitable reward connected to the routine and cue, the intervention is likely to be ineffective. This is of pertinence to animal welfare in connection with more challenging behaviours (e.g., giving wormer to a fearful horse) or individuals who may be resistant to change (e.g., someone who has slaughtered animals for many years), as identifying a motivating factor or long-term reward may be key in sustainably changing behaviour. However, studies have shown that motivations and rewards used when starting and continuing a behaviour do not always have to be overt or obvious in nature, which may go some way to addressing this challenge. This is an area for further consideration in relation to the development of PAWHBs.

Behaviour ‘change’ and ‘intention’ to change were evident during the study, with interviewees raising specific examples of changes that they had made or intended to make linked to the PAWHBInt. The study showed change in some of the participants, who communicated that the scratching behaviour had become automatic and that they were continuing to perform it, either with similar regularity or in relation to the contextual cue. Other participants found during the study that the horse or donkey preferred to be scratched in a different place than that scratched before the study, so intended to continue with the new scratching site, and yet others started scratching other equine animals in their care. Of interest were the two interviewees who did not receive positive feedback from the equine animal; both intended to continue with a positive interaction that was focused on quality time with the animal and their enjoyment together. Those interviewees who said that the behaviour was already a strong habit demonstrated a belief that this would remain the case.

### 4.3. Strengths and Limitations of the Study

This study is in a unique position as one of the first investigations into the application and use of human habit theory in an equine welfare context; its aim is to build a solid foundation to help address the current lack of research. As with any study, it is a process of evaluation and reflection, and a number of areas have been identified to aid future research in examining the potential of principles of human habit in sustainably improving animal welfare.

### 4.4. Participants

Owing to the restricted time available for recruiting participants, opportunistic sampling was used. This, together with the minimum size of group required for analysis, may have resulted in a sample of participants who were biased towards pro-equine welfare behaviour. While this provides useful insight into and understanding of existing PAWHBs, it was a limiting factor in assessing the value of the intervention in forming new PAWHBs, which resulted in the refocusing of the study design towards the qualitative research. In future work a randomised sample typical of the target population of interest should be used, with only participants who answer “no” in the before intervention survey being included in the remainder of the study. In addition, the majority of the participants were female, and while this may be representative of equestrian research, it indicates that increased male representation is needed in future studies. It should be noted that participants volunteered to undertake the study, this suggested that they were interested in the subject matter, which may have influenced how they responded to the PAWHBInt and the semi-structured interviews about their experience of the intervention. Recruiting a greater number of naïve participants at the outset will require more time but would address these concerns.

### 4.5. Language

It is recommended that the examples of scratching sites be removed to reduce the risk of demand characteristics from participants interpreting the aim of the study, as was identified in the qualitative analysis.

### 4.6. Self-Reporting

It is important to reflect on the limitations associated with self-report and recall, considering the persons perception of themselves, encoding and storage of memory, understanding what is required in the task, effective recall of what has happened, and determining how and what to communicate in an answer. Future research could include observational study and assessment of the behaviours to cross-check whether self-reported information was accurate.

### 4.7. Study Design and Materials

The design of the PAWHBInt was supported by data from the qualitative analysis (‘research insights’), with a simple approach (e.g., link to an existing routine) important for implementing any intervention in a practical equine welfare setting and in working with individuals resistant to change. The design provided informative data to aid future research. While the quantitative element of the study was not pursued owing to challenges with participant recruitment it did enable learning for future study design. This included the need to recruit naïve participants who were not performing or perceived they were performing the behaviour already, and ensuring that there was a gender balance among participants. The qualitative analysis was an important element of the study, as it facilitated greater insight into why people did or did not perform the PAWHB. These outcomes can be used to develop future research into the importance of human habit formation and change, together with supporting the development of practical interventions for sustainable improvements to animal welfare. Overall, the interview data indicated that the study materials used were understood by the participants (i.e., training materials, plan and SMD) and generally achieved their purposes.

### 4.8. Reflexivity

As with all qualitative research, the previous experience of those involved—whether the participants or those undertaking the investigation—will impact upon the findings. Therefore, it is important to take a reflexive approach. As outlined, the majority of participants were female, as was the PI, who was close to the average age, being 44 years old at the time of the study; she also provides care for equine animals. Her background of working in animal welfare, together with her explicit interest in increasing positive human–animal interactions, should be noted in relation to her view of the information communicated during the interviews and through the thematic analysis. To account for this the PI re-reviewed the data over a period of time, noting any emotional reactions or thoughts she had in response to the participants’ responses; in addition, she compared the study findings to those in other research and reflected on the meaning before finalising the themes.

### 4.9. Further Research and Recommendations

Further work is required to develop understanding around how human habit formation and change can benefit animal welfare. This includes examinations of habit in the context of theoretical concepts such as the habit loop (cue–routine–reward) [31], models such as the Theory of Interpersonal Behaviour [27] and evidence-based techniques such as Behaviour Change Technique Taxonomy (e.g., habit formation, habit reversal [p. 270] related to the COM-B Model [39]. There is a requirement to study specific elements of habit in relation to challenges in animal welfare (e.g., how to identify a reward that is valuable to participants involved in interventions).

Further reflection is required on the role of the non-human animal in human habit and its formation and adoption, together with the function of ‘mutual benefit’ and emotion. It is recommended that the initial focus should remain on behaviours that may be viewed positively by those who are the subject of the change in order to account for the reward element of the habit loop, which needs further exploration. It is suggested that this will provide greater understanding of habit theory applied in an animal-welfare context, before tackling behaviours that are more challenging in their nature and may represent a complicated route to reward (e.g., the handling of animals during slaughter;). These should be undertaken once there is a greater understanding of the practical and research-based application of habit theory to simple animal welfare routines.

It is clear that habit could have a valuable role to play in animal welfare through changing the behaviour of the human animals who interact with them. With many animal care behaviours being routine in nature and repeated in a consistent context (e.g., feeding, health checks, etc.), these lend themselves to habit formation, which presents an opportunity to develop pro-welfare care behaviours (e.g., weighing food before feeding to manage weight, routinely checking a horse for injuries each day). This research has also provided insight into the potential benefits to the human animal, such as emotional reward and positive feedback from the horses and donkeys they are interacting with. There is also a need to close the gap in knowledge in terms of its application both in research and practically in interventions. To do this effectively and produce the evidence required, further examination and consideration of suitable measurement tools and study design (e.g., observational) is required to establish how best to evaluate habit change. Future studies should consider a longitudinal design in order to test sustainability through repeated follow-ups over time. Finally, there is a need to match the examination of human habit formation or change to its impact upon animal welfare in order to determine whether it can make a lasting difference—as, ultimately, this is the question we need to answer.

## 5. Conclusions

Habit formation and change has untapped potential to positively and sustainably impact compromised welfare resulting from the absence of routine care-taking behaviours. This study found evidence of high levels of pre-existing PAWHBs among the participants and support for a PAWHBInt resulting in behaviour change or intention to change. These findings are in line with current research indicating that behaviour becomes easier to perform and less cognitively demanding the more times it is repeated. It generated support for the use of theory-based behaviour change interventions that link desired pro-animal welfare behaviour to an existing routine, in a consistent context, utilising repetition and supporting intervention features such as an action plan. The study results highlighted the importance of the ‘habit loop’ cue–routine–reward relationship in maintaining or forming a habitual behaviour, with emphasis placed upon the role of animal feedback in cueing the PAWHB and providing an emotional reward to the participants for performing it. While there is growing evidence in other sectors, there is a need to undertake further research to test and develop behaviour change interventions with habit at their centre in order to deliver positive change in the health and wellbeing of animals.

## Figures and Tables

**Table 1:** Overview of themes and the inquiry of their relation to the research question (*[31]). Abbreviations: HorCT—habit or conscious thought; RofR—role of routine; AF—animal feedback; C&I—change & intention; PAWHB—pro-animal welfare habitual behaviour;—pro-animal welfare habitual behaviour intervention.

Theme Description, Sub-Themes, Links to Other Themes	Insights into PAWHB: Maintained, Developed, Neither	Impact of Behaviour Change Technique of PAWHBInt
**Habit or Conscious Thought** (HorCT) (language indicating that the behaviour was, became, or was not habitual) **Sub-themes**: Developed & Learnt, Context **Links to other themes**: change, routine, research insights	Performing the PAWHB automatically: Yes, but performed the PAWHB before the study Yes, formed the PAWHB as a result of doing the study No, did not perform the behaviour automatically	Impact of the PAWHBInt on: Pre-existing established PAWHB PAWHB formed during the study Not forming the PAWHB or not continuing with the PAWHB after the study
**Role of Routine** (RofR) (language covering routine) **Sub-themes**: Action Plan, Memory, Consistent Context, Cue, Reminder, Repetition, Mutual Benefit, Emotion **Links**: HoCT, Change & Intention	The role of routine in maintaining, forming or not forming the PAWHB	The role and impact of linking the scratching behaviour to an existing routine as part of the PAWHBInt and other relevant elements of the intervention (e.g. action plan)
**Animal Feedback** (AF) (language covering the animal communicating interest/disinterest in the activity and the impact on the participant) **Sub-themes**: Cue, Mutual Benefit, Reward, Motivation, Emotion, Bonding, Context, Repetition, Willpower **Links**: RofR, HorCT	Indication that animal feedback is linked to habit maintenance, formation or non-formation, relating this to the ‘habit loop’ of cue–routine–reward *	Impact of animal feedback on the PAWHBInt linked to the ‘habit loop’
**Change & Intention** (C&I) (language indicative of change or intention to change) **Sub-themes**: Cue, Context, Emotion, Mutual Benefit, Learnt **Links**: HorCT, RofR, AF, Research Insights	Indications that the participants behaviour changed, or the participant communicated intention to change related to performing the PAWHB, either during or after the study	The role of the PAWHBInt in change or intention to change
**Research Insights** (language relating to the study and its design) **Sub-themes**: Human–Animal Interaction (*HAI*), Plan, Reminder, Training Materials, Cue, Reward, Motivation, SMD, Memory, Emotion **Links**: AF	Interesting findings, areas for future research that could assist the formation and maintenance of PAWHB	Interesting findings, areas for future research regarding the PAWHBInt

## Appendix A

Appendices will be placed on the Human Behaviour Change for Animals website (www.hbcforanimals.com) and assigned a URL that links to the article. In the meantime, they are available on request for the review process.
